# Determination of Hemicellulose, Cellulose and Lignin in Moso Bamboo by Near Infrared Spectroscopy

**DOI:** 10.1038/srep17210

**Published:** 2015-11-25

**Authors:** Xiaoli Li, Chanjun Sun, Binxiong Zhou, Yong He

**Affiliations:** 1 College of Biosystems Engineering and Food Science, Zhejiang University, 866 Yuhangtang Road, Hangzhou 310058, China

## Abstract

The contents of hemicellulose, cellulose and lignin are important for moso bamboo processing in biomass energy industry. The feasibility of using near infrared (NIR) spectroscopy for rapid determination of hemicellulose, cellulose and lignin was investigated in this study. Initially, the linear relationship between bamboo components and their NIR spectroscopy was established. Subsequently, successive projections algorithm (SPA) was used to detect characteristic wavelengths for establishing the convenient models. For hemicellulose, cellulose and lignin, 22, 22 and 20 characteristic wavelengths were obtained, respectively. Nonlinear determination models were subsequently built by an artificial neural network (ANN) and a least-squares support vector machine (LS-SVM) based on characteristic wavelengths. The LS-SVM models for predicting hemicellulose, cellulose and lignin all obtained excellent results with high determination coefficients of 0.921, 0.909 and 0.892 respectively. These results demonstrated that NIR spectroscopy combined with SPA-LS-SVM is a useful, nondestructive tool for the determinations of hemicellulose, cellulose and lignin in moso bamboo.

Bamboo is considered as one of the most important lignocellulosic biomass, due to its rapid growth, high productivity, low ash content and alkali index^1^, it has great potential to be used as a sustainable feedstock for fuel ethanol production^2^. Bamboo is distributed mostly in Asia, especially in the tropics and subtropics. According to estimates, Asia has more than 1.8 × 10^7^ ha of bamboo, which is over 80% of the world’s total^3^. Moso bamboo is the most widely distributed bamboo in China, accounting for 65% of the total area of bamboo forest^4^. The main chemical compositions of moso bamboo are hemicellulose, cellulose and lignin which make up more than 90% of the total dry mass. And the sum of hemicellulose and cellulose is about 65%^5^. In the utilization of bamboo in biomass energy, the chemical composition and structure could have a significant effect on reactivity during chemical and enzymatic pretreatment, hydrolysis and fermentation. And, the chemical components of bamboo obtained from different locations or physiological ages may show varied contents, as well as within an individual bamboo culm. Hence, the real-time monitoring of the components is of great importance for the optimization of biomass process.

The Van Soest method^6^ is a traditional wet chemistry method for measurement of the hemicellulose, cellulose and lignin. In fact, the traditional wet chemistry methods are time consuming, chemical reagent consuming and laborious, which can’t meet the requirement of rapid and real-time detection in large-scale industrial biomass utilization^7^. So, further research for rapid determination of components of bamboo is required.

Recently, visible and near infrared (NIR) spectroscopy has been recognized as one of the most promising technique for prediction of physical and chemical properties of mass materials, due to its powerful, rapid, nondestructive, simple sample preparation and good reproducibility^8^. NIR has been used in analyzing compositions of wood, corn stover, and rice straw^8^, such as lignin^9^,^10^, cellulose^11^,^12^ and hemicellulose^8^. Meanwhile, NIR spectroscopy has also been applied for measuring chemical components of bamboo, such as Klason lignin contents^13^,^14^, neutral detergent fiber (NDF), acid detergent fiber (ADF)^15^ and holocellulose, a-cellulose^14^. But, a deep NIR investigation of bamboo need to be conducted to enhance the determination accuracy and stability of model. In general, the reported NIR studies of bamboo were all based on linear calibration model of partial least squares (PLS) regression. Although PLS is an effective algorithm for linear modeling, it could not accurately express the nonlinear relationship that usually exists in spectra analysis^16^. Meanwhile, non-linear calibration algorithm can employ both of linear and non-linear relationships into the determination model for a higher accuracy. Recently, a powerful tool called least-squares support vector machine (LS-SVM)^17^,^18^ has been reported in NIR spectral analysis^19^, and it is especially good at modeling the spectra data characterized by nonlinearity, small samples, and high dimension^16^. However, related research focused on bamboo was scarce.

The ability of NIR for analysis of components of biomass relies on the selective absorption of light by the overtones and combinations vibrations of C-O, O-H, C-H and N-H bonds in chemical compounds^20^,^21^, which generates broad and highly overlapping NIR absorption peaks. So it is difficult to directly relate distinct absorption band to the chemical components of biomass^21^. The reported NIR studies on biomass almost adopted full range spectra or hundreds of continuous spectral range as independent variables. It is worth noting that reducing the dimension of independent variables is critical to improve the performance of the NIR predictive models for timely decision making in engineering applications such as online assessment of biomass^7^. Moreover, the reduction of variables dimension is also helpful to recognize and select characteristic bands for modeling, because it not only provides simply interpretation but also saves the cost of determination^8^. Recently, several studies summarized characteristic absorption bands corresponding to the functional groups in biomass materials^8^,^9^, which will greatly deepen the understanding of the relationship between NIR signal and chemical components of biomass. However, the reported NIR studies on biomass almost adopted full range spectra or hundreds of continuous spectral range as independent variables, the potential of characteristic bands hasn’t been proved by quantitative modeling.

In this manuscript, the potential of non-linear calibration algorithm of radial basis function neural network (RBF-NN) and LS-SVM were investigated for establishment of accurate and robust model for bamboo components, and the performance and robustness of non-linear models were compared with the linear model of PLS. Moreover, characteristic wavelengths were recognized and selected for developing more convenient prediction models, and a comparison of the characteristic wavelengths models and full spectra model was conducted.

## Results and Discussion

### NIR spectra analysis

**Figure 1:**
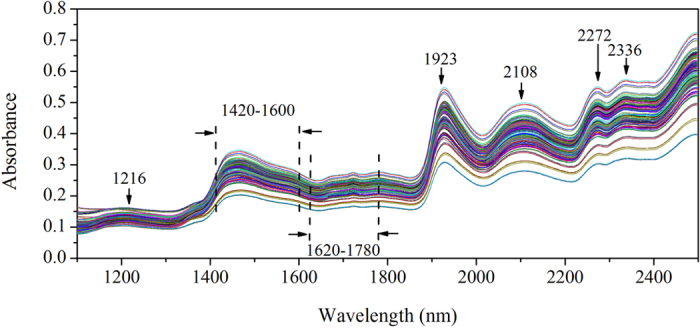
Spectrogram of the bamboo powder.

As mentioned in the introduction, NIR spectra can provide the structural information of components of biomass by analyzing the broad and highly overlapping NIR absorption peaks. The main absorption peaks of bamboo powder were marked in Fig. 1. As seen in Fig. 1, a weak and broad absorption peak around 1216 nm is assigned to C–H stretching (2nd overtone)^14^,^22^. Absorptions in the region of 1420–1600 nm are associated with O–H stretching (1st overtone)^23^. The signal in the region of 1620–1780 nm are attributed to C–H groups (1st overtone)^14^,^24^. The band at approximately 1923 nm is attributable to –OH and –C = O groups^25^. The signal at around 2108 nm is ascribed to the combination of O–H and C–H stretching vibrations^26^. The absorption peak at approximately 2272 nm is assigned to the combination bands of O–H and C–O^8^. The peak at 2336 nm belongs to C–H stretching and deformation group frequencies of polysaccharides^21^.

### Establishment of the linear determination models

Hemicellulose, cellulose and lignin contents vary across the different samples and only the robust variation tendencies were observed in practice, which cannot meet the requirement of accurate determination. Therefore, linear determination models combined with spectral information and chemical values were developed for the rapid and real-time detections of hemicellulose, cellulose and lignin contents of moso bamboo.

### Regression models based on PLS

The analytical information of NIR spectra is often influenced by light scattering, noise signal and baseline drift, which are produced during the operational process. These influence factors will have adverse effects on the accuracy of the detection models. Therefore, before establishing the detection models, the spectral data were first pretreated by five pretreatments to reduce as much as interference information. These pretreatments were smoothing (SM), multiplicative scatter correction (MSC), first derivate (1st DER), second derivate (2nd DER) and wavelet transform (WT). Then, 114 samples in the calibration set were used to build PLS models on the full spectral range (1100–2498 nm) for hemicellulose, cellulose and lignin. The modeling results based on different pretreatments are shown in Table 1. As mentioned in section 2.6, coefficient of multiple determination for calibration (Rc^2^), coefficient of multiple determination for prediction (Rp^2^), standard error of calibration (SEC), standard error of prediction (SEP) and residual predictive deviation (RPD) are five important indicators in model evaluation. R_c_^2^/ R_p_^2^ values close to 1, SEC/SEP values close to 0 and a high RPD value, as well as small differences between the calibration and prediction sets, indicate a better fit. As seen in Table 1, it can be found that the prediction models of hemicellulose, cellulose and lignin based on the original spectra all obtained good results with R_p_^2^ higher than 0.82, and RPD bigger than 2.3, indicating that NIR spectroscopy is a power tool for determination of hemicellulose, cellulose and lignin of bamboo. However, there were obvious differences of model performances between calibration and prediction sets, this phenomenon may be due to the disturbance of the noise signal and the collinearity of spectroscopic data. After pretreatments, the performances of these models fluctuated, among which the one based on WT pretreatment obtained the optimal results. The model performances of hemicellulose and lignin pretreated by WT were improved, and Rp^2^ were improved from 0.841 and 0.824 to 0.842 and 0.835, respectively. As for the model of cellulose pretreated by WT, Rp^2^ was comparable with that based on the original data. Moreover, the differences of model performances between calibration and prediction sets decreased through WT pretreatment. In general, WT improved the performances of these detection models and the good performance of these models pretreated by WT indicated that WT was useful in eliminating noise signal and reducing collinearity of the spectral data. Thus, the data pretreated by WT was used for further analysis.

### Selection of characteristic wavelengths based on SPA

**Figure 2:**
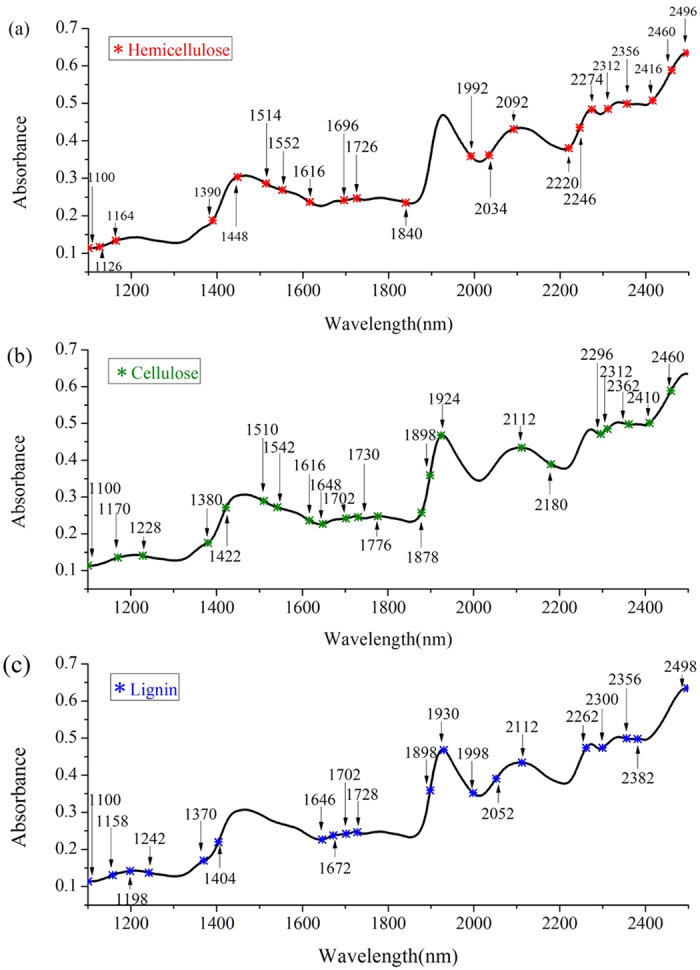
Distributions of the characteristic wavelengths selected by SPA for hemicellulose (a), cellulose (b) and lignin (c).

To enhance the accuracy and convenience of the models for real determination, further optimization of independent variables was performed. The successive projections algorithm (SPA) was proposed to select the most sensitive wavelengths for the determination of hemicellulose, cellulose and lignin. And 22, 22 and 20 characteristic wavelengths were selected by SPA for hemicellulose, cellulose and lignin, respectively. The distributions of the characteristic wavelengths are shown in Fig. 2, and the multivariate linear regression (MLR) modeling results based on these characteristic wavelengths are shown in Table 2.

Generally speaking, absorptions at the selected characteristic wavelengths are closely associated with the structures of the chemical components. As seen in Fig. 2, a number of characteristic wavelengths, marked above the spectral lines, are shared by hemicellulose, cellulose and lignin, which demonstrated that parts of the structures were similar among the three components. The characteristic wavelengths around 1380 nm, shared by hemicellulose, cellulose and lignin, correspond to C–H stretching and deformation of –CH_3_^21^. Absorptions in the region of 1400–1660 nm are associated with O–H stretching (1st overtone) for hemicellulose and cellulose^13^. As for lignin, the characteristic wavelength of 1404 nm is connected with O–H stretching (1st overtone) and the wavelengths of 1646 nm, 1672 nm and 1702 nm are connected with C–H vibration^13^. The signal approximately 1725 nm, which appears in the shared characteristic wavelengths for hemicellulose, cellulose and lignin, may relate to the C–H stretching (1st overtone) of –CH_2_^21^. The common characteristic wavelengths of 1898 nm and approximately 1927 nm for cellulose and lignin are ascribed to C = O stretching (2nd overtone) of –CO_2_H^21^ and the combination of O–H stretching and deformation vibrations^26^, respectively. The shared wavelength around 1996 nm for hemicellulose and lignin may correspond to the combination of O–H stretching and C = O stretching (2nd overtone)^22^. The signal around 2100 nm is connected with the combination of O–H and C–H stretching vibrations for hemicellulose, cellulose and lignin^26^. Wavelengths approximately 2280 nm and 2322 nm of hemicellulose, cellulose and lignin belong to C–H stretching and deformation group frequencies^21^.

As seen in Table 2, Rc^2^, Rp^2^ and RPD for hemicellulose and lignin were relatively lower than that with the full spectral range pretreated by WT, indicating the performances of these models based on the characteristic wavelengths were slightly worse than that with the full spectral range. This leaded to the conclusion that the accuracies of the models for hemicellulose and lignin were decreased by reduction of the independent variables with SPA. However, as for cellulose, the situation was different. As seen in Table 2, Rc^2^, Rp^2^ and RPD of the detection model based on the characteristic wavelengths for cellulose were higher than that with the full spectral range, which indicated that SPA was useful in improving the accuracy of the detection model for cellulose. Furthermore, the most remarkable facet of the SPA used in this study was the reduction of the independent variables from 700 to 22, 22 and 20 for hemicellulose, cellulose and lignin, respectively. And this reduction greatly simplified the structure of determination model, promoted the detection efficiency, and would contribute to developing simple and low-cost instruments. Thus, nonlinear algorithms were proposed to establish model with high accuracy.

### Establishment of the nonlinear determination models

Linear determination models for hemicellulose, cellulose and lignin have been established by combining SPA and MLR. However, a nonlinear relationship that generally exists in spectral analysis cannot be expressed by MLR. Thus, RBF-NN and LS-SVM were proposed to explore the nonlinear relationship between spectral information and chemical compositions.

### Regression models based on RBF-NN

Considering the good performances of the characteristic wavelengths selected by SPA, these wavelengths were used as independent variables to develop RBF-NN model. Thus, the spectral information of the 22, 22, 20 characteristic wavelengths were set as the input variables to build RBF-NN models for hemicellulose, cellulose and lignin, respectively. Spread is an important parameter influencing the performance of any neural network. If the spread is too small, convergence of the network may be prevented; however, if it is too large, overtraining of the network may result. Therefore, the spread values for hemicellulose, cellulose and lignin were first optimized. The spread ranges for the hemicellulose, cellulose and lignin regression models were all set as 100–2500. Through double training cycles of the network, the optimal spread values were selected according to the minimal RMSE values of the prediction set. The optimal spread values were eventually determined to be 948, 126 and 1254 for hemicellulose, cellulose and lignin, respectively. The results of the RBF-NN models are shown in Table 3.

As seen in Table 3, the nonlinear models based on RBF-NN obtained Rp^2^ values of 0.807, 0.891 and 0.780 for hemicellulose, cellulose and lignin, respectively. Comparing with the linear model (shown in Table 2), the RBF-NN models obtained better predictive performance with higher Rp^2^ values, which demonstrated that the nonlinear relationship between spectral information and chemical compositions was expressed to a certain extent by RBF-NN. However, comparing with the results of PLS models based on the full spectral range pretreated by WT, the results of RBF-NN models for hemicellulose and lignin were still less well-performed. Therefore, the results of nonlinear models should be further improved.

### Regression models based on LS-SVM

**Figure 3:**
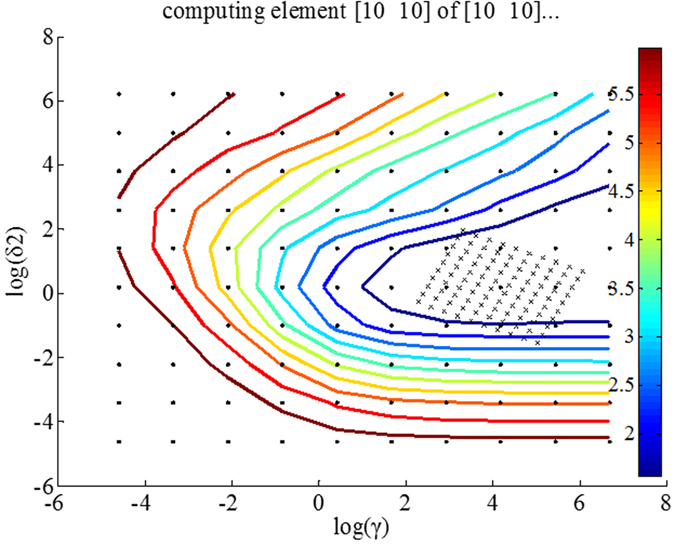
Optimization of γ and δ^2^ in building the LS-SVM model for hemicellulose.

LS-SVM was proposed to improve the nonlinear models. The spectral information at the characteristic wavelengths were regarded as independent variables and the corresponding chemical values served as dependent variables. Meanwhile, a radial basis function (RBF) was used as a kernel function. Two main parameters (γ and δ^2^) were first determined before building the LS-SVM model. The penalty factor (γ) not only balances the structural and empirical risk minimizations in the model but also plays an important role in improving the generalization of the model. The width of the kernel function (δ^2^) controls the regression error of the model and reflects the sensitivity imparted by the input variables. Only when the appropriate parameters are selected will the accuracy of the model prediction be ensured. In this study, the grid searching technique was used to optimize the two parameters. The ranges of γ and δ^2^ for hemicellulose, cellulose and lignin were set according to previous experiments and shown in Table 4. The searching procedures for the optimal γ and δ^2^ values for hemicellulose (taken as an example) are shown in Fig. 3.

**Figure 4:**
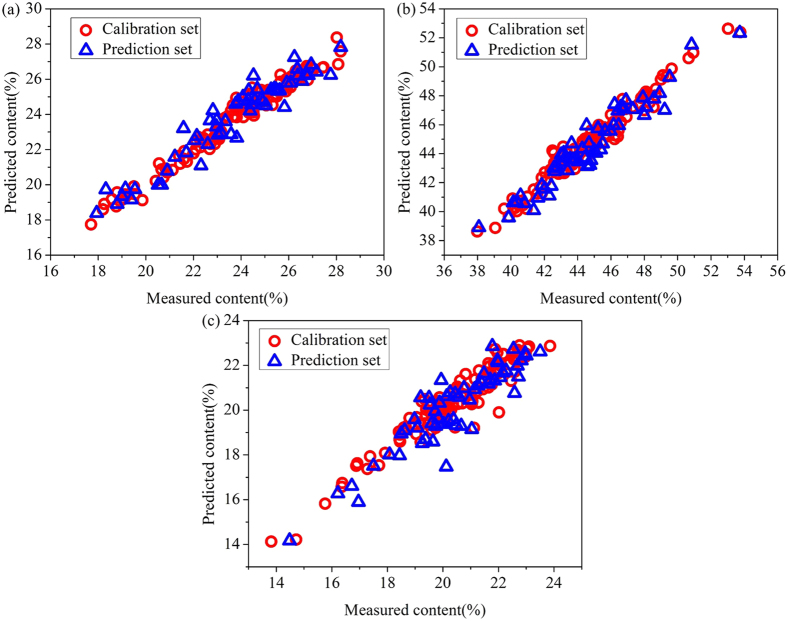
LS-SVM graphs of predicted versus measured values for hemicellulose (a), cellulose (b) and lignin (c) based on characteristic wavelengths.

As seen in Fig. 3, the process of optimization consisted of two steps: coarse screening and fine screening. The grid points in coarse screening were 10 × 10, represented by “■”. The optimal range is represented by the contour plot of error. Fine screening was built on the basis of the coarse screening as shown above. The grid points were also 10 × 10, represented by “×”. The step size was much smaller than in coarse screening. The final results of the LS-SVM models for hemicellulose, cellulose and lignin are summarized in Table 4 and the distributions of the predicted versus measured values are shown in Fig. 4.

As seen in Table 4, changes in the determination components led to the choice of different optimal parameters of γ and δ^2^. Comparing with the RBF-NN models based on the characteristic wavelengths and the linear models based on the full spectral range, all the performances of the LS-SVM models were greatly enhanced, with Rc^2^ values above 0.940, Rp^2^ values roughly 0.900, SEC values lower than 0.600 and SEP values lower than 0.900. Meanwhile, the RPD values of hemicellulose, cellulose and lignin models were all greater than 3. In general, on the basis of independent variables simplification, the LS-SVM models obtained wonderful prediction results with high fitting degrees and measurement accuracies, which can also be seen in Fig. 4 intuitively.

Sun *et al.*^14^ collected the FT-NIR spectra of 90 bamboo samples with the spectral range of 350–2500 nm and established PLS determination models for holocellulose, α-cellulose and Klason lignin, respectively, the results were prediction R^2^ of 0.91, RMSEP of 1.05% and RPD of 3.18 for holocellulose; prediction R^2^ of 0.97, RMSEP of 0.81% and RPD of 5.42 for α-cellulose; prediction R^2^ of 0.66, RMSEP of 0.65% and RPD of 1.62 for Klason lignin. Huang *et al.*^13^ built a PLS model for determination of Klason lignin in bamboo based on 53 samples at the wavelength range of 1100–2500 nm, and obtained prediction R^2^ of 0.93, SEP of 0.66 and RPD of 3.72. Comparing with the results of holocellulose and α-cellulose performed by Sun *et al.* and that of Klason lignin performed by Huang *et al.*, the models based on LS-SVM in this research obtained comparable R^2^ and RPD values. While, compared with the results of Klason lignin executed by Sun *et al.*, the prediction R^2^ increased from 0.66 to 0.892 and RPD increased from 1.62 to 3.129, which indicated that the nonlinear determination models could enhance the accuracy and precision of the prediction results. Moreover, for the sample size, this research expanded the relatively small samples (90 samples in Sun *et al.*^14^ and 53 samples in Huang *et al.*^13^) to 171 samples, which greatly improved the representativeness of the samples, making the applicability of the models stronger. Furthermore, the independent variables used in this study were greatly reduced to 22, 22 and 20 for hemicellulose, cellulose and lignin by wavelength selection, which were less than that used by Sun *et al.*^14^ (378 variables in the spectral range of 350–2500 nm) and Huang *et al.*^13^ (140 variables in the spectral range of 1100–2500 nm), this reduction significantly simplified the determination models, accelerated the testing speed and improved the working efficiency. By extension, the reduced independent variables will contribute to further development of convenient and low-cost online measuring device.

## Conclusions

This research explored the feasibility of NIR spectroscopy for determination of hemicellulose, cellulose and lignin in moso bamboo. SPA was proposed to recognize characteristic wavelengths, which were closely related with hemicellulose, cellulose and lignin. The LS-SVM models based on these characteristic wavelengths outperformed the models based on SPA-MLR and SPA-RBF-NN, obtaining prediction R^2^ values of 0.921, 0.909 and 0.892 for hemicellulose, cellulose and lignin, respectively. As a whole, the feasibility of NIR spectroscopy for rapid determination of cellulose, hemicellulose and lignin in moso bamboo was proved, and models based on SPA-LS-SVM may provide important guidance for bamboo biomass energy industry.

## Materials and Methods

### Sample collection

The moso bamboo samples were taken from three different locations: Maoyang Village, Jingning County, Zhejiang Province, China (27°43′S, 119°23′E); Baitanao Village, Jingning County, Zhejiang Province, China (27°49′S, 119°19′E); Guangan County, Sichuan Province, China (30°27′S, 106°38′E). These were denoted as A, B and C, respectively. At each harvest location, bamboos aged from 1 year to 5 years were obtained. To expand range of natural variability, four positions of each bamboo culm were taken as samples. The positions were divided, in detail, as following: the second bottom bamboo internode (marked with a), the middle bamboo internode (marked with b), the second top bamboo internode (marked with c) and the bamboo joints of these locations (marked with d). Three repetitions were taken in each type and a total of 180 samples were investigated in this study.

### Sample preparation

After harvesting, the bamboos were air-dried. The dried bamboos were then split into canes and cut into pieces. Subsequently, the bamboo pieces were milled by a grinder (Tissuelyser-48, Shanghai, China). The bamboo powder was sifted through screens with mesh widths of 380 μm and 250 μm. The sieved powder with particle sizes between 380 μm and 250 μm was collected for further analysis.

### NIR spectroscopy collection

NIR spectra of the powder samples were acquired on a FOSS NIR Systems 5000 spectrometer (Silver Spring, MD, USA). The spectra were collected in the wavelength range of 1100–2498 nm. The data were saved as log (1/R), where R represents the diffuse reflectance. Each sample was scanned 3 times by successive rotation with an angle of 120°. The average spectrum was regarded as the sample spectrum. A software of Winscan v1.50 was used for the spectral measurement and analysis.

### Chemical experiments

The hemicellulose, cellulose and lignin contents were detected by the traditional Van Soest method^6^. Bamboo powder of 0.50 g was accurately weighed for the chemical measurement. All of the reagents used in this study were of analytical grade. The relative error of the three repeated chemical measurements of each sample was controlled lower than 5%.

### Elimination of abnormal samples and sample division

**Figure 5:**
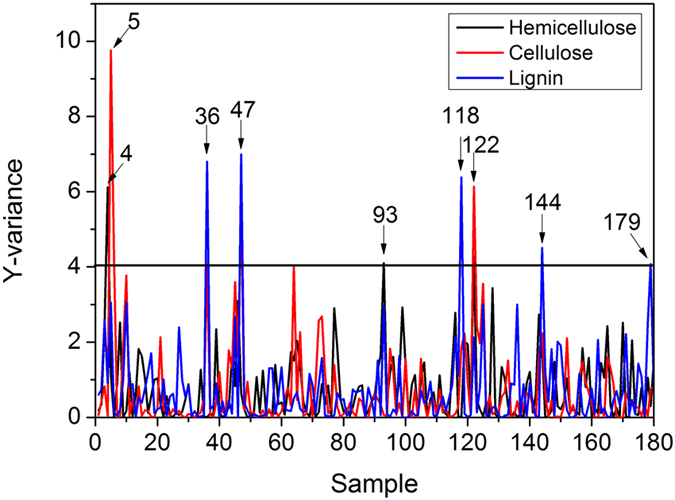
Y-variance of the samples in PLS models.

Abnormal samples will seriously decrease the precision of the prediction model. A partial least squares (PLS) regression method was used to recognize abnormal samples. All 180 samples were first used to build the PLS regression models over the entire wavelength range (1100–2498 nm) for hemicellulose, cellulose and lignin. As seen in Fig. 5, 9 samples (4, 5, 36, 47, 93, 118, 122, 144 and 179) with high Y-variance values, were regarded as abnormal samples and were eliminated in the following process.

To fully evaluate the determination model, the samples were divided into two sets. Firstly, the samples were sorted according to increasing chemical content values, and the median of every three was selected for the prediction. The remaining samples were obtained for the calibration set. There were 114 and 57 samples in the calibration and prediction set, respectively. Meanwhile, full cross validation was used to verify the accuracy of the model. The statistical analysis of the sample division is shown in Table 5.

### Chemometric analysis

The PLS algorithm is a multivariate statistical analysis method, widely used in spectral analysis. Through use of the PLS, comprehensive information can be obtained by maximizing the variance of the main components. The linear relationship between the spectral information and the chemical composition values is used for determining the maximal degree of correlation^27^. In this study, PLS was used to eliminate abnormal samples for hemicellulose, cellulose and lignin. And the PLS was implemented based on the Unscrambler V9.8 (Camo, Process, AS, Oslo, Norway), a multivariate statistical and analytical software package.

NIR spectra is often affected by factors such as background noise, light scattering, and the inhomogeneity of the sample. Therefore, proper pretreatments of the spectral information are usually needed to remove the effects of interference factors^28^. In this research, the following methods were applied to pretreat the data: Savitzky-Golay smoothing (SM)^29^, multiplicative scatter correction (MSC)^30^, Savitzky-Golay first derivative (1st DER)^31^, Savitzky-Golay second derivative (2nd DER)^31^ and wavelet transform (WT)^32^. SM is often used to smooth the noisy signal by fitting a polynomial to the spectral data^33^. MSC is aimed to reduce the scattering interferences of particle size^34^. DER is attempted to eliminate the baseline offset variations^35^. WT is commonly used to remove the noisy signal by transforming the original spectral information into the wavelet domain^36^. The pretreatment computations of SM, MSC, 1st DER and 2nd DER were implemented based on the Unscrambler V9.8 (Camo, Process, AS, Oslo, Norway), and the WT was conducted in the Matlab R2010b (The MathWorks, Natick, MA, USA).

The successive projections algorithm (SPA) is a method used for the selection of sensitive wavelengths. The variable set with the minimum redundancy is selected from the spectral information, effectively eliminating collinearity between variables with the least number of variables^37^. Details of the SPA algorithm are shown in the literature^38^. The SPA was proposed here to minimize the complexity of the linear determination model, making a convenient and rapid determination of the hemicellulose, cellulose and lignin contents in bamboo, especial for rapid real-time measurement. The SPA was implemented by the software of gui_spa provided by Araújo *et al.*^38^ and the detailed calculations was performed by homemade codes in Matlab R2010b (The MathWorks, Natick, MA, USA).

The radial basis function neural network (RBF-NN) is a feed-forward network, which has been proved to approximate continuous functions in an arbitrary precision with the best approximation^39^. Furthermore, the convergence speed of the RBF-NN is faster than that of the global approximation network^39^. Details of the RBF-NN algorithm are shown in the literature^40^. In this research, RBF-NN was performed to build nonlinear determination models for the hemicellulose, cellulose and lignin contents in bamboo. RBF-NN was operated in the Matlab R2010b (The MathWorks, Natick, MA, USA).

A support vector machine (SVM) is a general learning method developed on the basis of statistical learning theory. Its basic idea is derived from an optimal separating hyperplane, which requires that the hyperplane not only separate two classes of samples but also maximizes the classification space^41^. A least squares support vector machine (LS-SVM) is an extension of SVM^16^. This method transfers inequality constraints into equality constraints, thereby reducing the computational complexity and is quite suitable for a small sample sizes, nonlinear systems and high dimensional data sets^42^. Here, the method was also used in attempts to build nonlinear determination models for the hemicellulose, cellulose and lignin contents in bamboo. LS-SVM was operated in the Matlab R2010b (The MathWorks, Natick, MA, USA) combining with LS-SVM toolbox (LS-SVM v 1.5, Suykens, Leuven, Belgium).

There are five important indicators in the evaluation of the model performance: coefficient of multiple determination for calibration (R_c_^2^), coefficient of multiple determination for prediction (R_p_^2^), standard error of calibration (SEC), standard error of prediction (SEP) and residual predictive deviation (RPD). The definitions of R^2^, SEC, SEP and RPD are shown as follows.

R^2^ (R_c_^2^/ R_p_^2^) measures how successful the fit is in explaining the variation of the data. A value close to 1 indicates a good fit. R^2^ is calculated as:


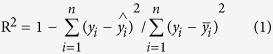


_Where *n* is the number of samples,_ 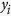
_is the true chemical value for the *i*th sample,_ 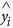
_is the predicted chemical value for the *i*th sample,_ 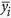
_is the mean of_ 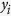
_for all the samples._

SEC and SEP reflect the precision of the measurement, with values close to 0 indicating a good fit. The formulas for determining SEC and SEP are shown as follows:


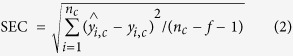


_Where_ 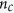
_is the number of samples in calibration set,_ 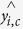
_is the predicted chemical value for the *i*th sample in calibration set,_ 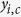
_is the true chemical value for the *i*th sample in calibration set,_ 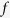
_is the number of variables used in the regression equation._


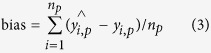



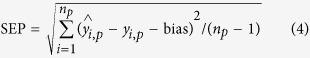


_Where_ 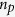
_is the number of samples in prediction set,_ 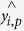
_is the predicted chemical value for the *i*th sample in prediction set,_ 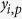
_is the true chemical value for the *i*th sample in prediction set._

RPD is calculated to assess the predictive ability of the NIR model^14^. The higher value of the RPD is, the more powerful of the predictive ability the model obtains^43^. In specific agricultural application, an RPD more than 1.5 is regarded good for preliminary screenings and initial predictions^44^; an RPD between 2.0 and 2.5 is considered satisfactory for prediction^20^; an RPD greater than 3.0 indicates that the model could predict efficiently^45^. RPD is calculated as:


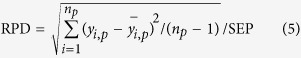


_Where_ 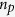
_is the number of samples in prediction set,_ 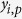
_is the true chemical value for the *i*th sample in prediction set,_ 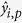
_is the mean of_ 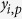
_for all the samples in prediction set._

## Additional Information

**How to cite this article**: Li, X. *et al.* Determination of Hemicellulose, Cellulose and Lignin in Moso Bamboo by Near Infrared Spectroscopy. *Sci. Rep.* **5**, 17210; doi: 10.1038/srep17210 (2015).

## Figures and Tables

**Table 1:** Results of the PLS models for hemicellulose cellulose and lignin with different pretreatments based on the full spectral range.

Pretreatment		ORI	SM	MSC	1^st^ DER	2^nd^ DER	WT
Hemicellulose	R_c_^2^	0.911	0.897	0.904	0.755	0.680	0.904
	SEC (%)	0.757	0.815	0.787	1.255	1.434	0.784
	R_p_^2^	0.841	0.838	0.835	0.535	0.518	0.842
	SEP (%)	1.016	1.025	1.033	1.736	1.754	1.014
	RPD	2.511	2.493	2.472	1.470	1.457	2.518
Cellulose	R_c_^2^	0.867	0.866	0.864	0.962	0.980	0.867
	SEC (%)	1.058	1.060	1.069	0.563	0.412	1.058
	R_p_^2^	0.834	0.833	0.832	0.755	0.342	0.834
	SEP (%)	1.192	1.197	1.199	1.446	2.365	1.192
	RPD	2.447	2.437	2.432	2.027	1.233	2.447
Lignin	R_c_^2^	0.940	0.924	0.935	0.936	0.895	0.926
	SEC (%)	0.450	0.509	0.471	0.466	0.599	0.501
	R_p_^2^	0.824	0.832	0.832	0.601	0.474	0.835
	SEP (%)	0.769	0.750	0.751	1.150	1.320	0.744
	RPD	2.390	2.497	2.493	1.599	1.418	2.516

**Table 2:** Results of MLR models for hemicellulose, cellulose and lignin based on the characteristic wavelengths.

Component	R_c_^2^	SEC (%)	R_p_^2^	SEP (%)	RPD
Hemicellulose	0.899	0.805	0.789	1.152	2.218
Cellulose	0.934	0.742	0.888	0.980	2.977
Lignin	0.890	0.612	0.767	0.836	2.240

**Table 3:** Results of RBF-NN models for hemicellulose, cellulose and lignin based on the characteristic wavelengths.

Component	R_c_^2^	SEC (%)	R_p_^2^	SEP (%)	RPD
Hemicellulose	0.891	0.834	0.807	1.112	2.298
Cellulose	0.936	0.729	0.891	0.961	3.035
Lignin	0.860	0.687	0.780	0.855	2.189

**Table 4:** Results of LS-SVM models for hemicellulose, cellulose and lignin based on characteristic wavelengths.

Component	Range of γ	Optimal γ	Range of δ^2^	Optimal δ^2^	R_c_^2^	SEC (%)	R_p_^2^	SEP (%)	RPD
Hemicellulose	1–1.000 × 10^6^	6.779 × 10^5^	1–1.000 × 10^6^	3.388 × 10^2^	0.982	0.340	0.921	0.710	3.598
Cellulose	1–2.000 × 10^7^	1.139 × 10^7^	1–1.000 × 10^5^	5.573 × 10^3^	0.959	0.585	0.909	0.876	3.328
Lignin	1–5.000 × 10^2^	1.510 × 10^2^	1–3.000 × 10^3^	9.391	0.947	0.422	0.892	0.598	3.129

**Table 5:** Statistical analysis of samples in the calibration and prediction sets.

Set	N	Hemicellulose				Cellulose				Lignin			
		Max (%)	Min (%)	Ave (%)	SD (%)	Max (%)	Min (%)	Ave (%)	SD (%)	Max (%)	Min (%)	Ave (%)	SD (%)
Cal	114	28.18	17.70	23.65	2.53	53.76	37.98	44.63	2.90	23.86	13.82	20.35	1.85
Pre	57	28.18	19.94	23.65	2.55	53.72	38.08	44.64	2.93	23.50	14.48	20.36	1.84
Total	171	28.18	17.70	23.65	2.53	53.76	37.98	44.64	2.90	23.86	13.82	20.35	1.84
